# Therapeutically targeting the unique disease landscape of pediatric high-grade gliomas

**DOI:** 10.3389/fonc.2024.1347694

**Published:** 2024-03-08

**Authors:** Dasun Fernando, Afsar U. Ahmed, Bryan R. G. Williams

**Affiliations:** ^1^ Centre for Cancer Research, Hudson Institute of Medical Research, Monash University, Clayton, VIC, Australia; ^2^ Department of Molecular and Translational Sciences, Faculty of Medicine, Nursing and Health Sciences, Monash University, Clayton, VIC, Australia

**Keywords:** cancer, oncology, pediatric high-grade glioma (pHGG), diffuse midline glioma (DMG), precision medicine & genomics, immunotherapy, nanomedicine, blood-brain barrier

## Abstract

Pediatric high-grade gliomas (pHGG) are a rare yet devastating malignancy of the central nervous system’s glial support cells, affecting children, adolescents, and young adults. Tumors of the central nervous system account for the leading cause of pediatric mortality of which high-grade gliomas present a significantly grim prognosis. While the past few decades have seen many pediatric cancers experiencing significant improvements in overall survival, the prospect of survival for patients diagnosed with pHGGs has conversely remained unchanged. This can be attributed in part to tumor heterogeneity and the existence of the blood-brain barrier. Advances in discovery research have substantiated the existence of unique subgroups of pHGGs displaying alternate responses to different therapeutics and varying degrees of overall survival. This highlights a necessity to approach discovery research and clinical management of the disease in an alternative subtype-dependent manner. This review covers traditional approaches to the therapeutic management of pHGGs, limitations of such methods and emerging alternatives. Novel mutations which predominate the pHGG landscape are highlighted and the therapeutic potential of targeting them in a subtype specific manner discussed. Collectively, this provides an insight into issues in need of transformative progress which arise during the management of pHGGs.

## 1 Introduction

The global pediatric cohort accounts for a rare yet significantly vulnerable societal population in terms of overall cancer incidence (1). The pediatric population is generally defined to encompass infants, children and adolescents in a clinical setting. While birth (age 0) is universally accepted as a valid starting time point for one to be clinically considered as pediatric, discrepancies exist among different countries when defining the cut-off age for pediatric consideration, and this has proved to be a source of contention for pediatric oncologists (2–5). For instance, the maximum ages for pediatric care in Australia, the United States and the United Kingdom are 22, 21 and 18 years of age respectively. However, the general global consensus can be summarized by the ages 18-22 being the upper limit for pediatric clinical care (2–5). Recent studies have illustrated central nervous system (CNS) tumors of the brain and spinal cord as accounting for approximately 15% of such pediatric cancer cases, ranking second for prevalence behind leukemia (approximately 35% of cases) (6). One third of these CNS tumors arise between the ages 0 and 4 (6). Despite having a much lower prevalence than leukemia, pediatric CNS tumors are the leading cause of childhood cancer mortality with 40% of deaths attributed to the disease (1–7). Advances in research and clinical understanding over the past 30 years have elevated the 5-year life expectancy of childhood cancer patients from 71% to 84%, with leukemia experiencing a significant 20% increase. Contrariwise, CNS tumors have remained stagnant with a mere increase of 4%, emphasizing an urgent need to accelerate research and clinical progress (1–7).

Pediatric high-grade gliomas (pHGG) are a debilitating malignant tumor of heterogenous nature, which originate from the glial cells of the brain (8). Glial cells of the CNS including astrocytes, oligodendrocytes, microglia and ependymal cells, serve a pivotal role as the support cells of the brain with primary functions being to provide structural integrity to neurons and the blood-brain barrier (BBB), myelination, oxygen, nutrients, synthesize and circulate cerebrospinal fluid (CSF) and conduct immunosurveillance. Tumorigenic derivatives arising from glial cells are referred to as gliomas, the most predominant form of pediatric brain tumor diagnosed annually (9). As of 2021, the World Health Organization (WHO) has defined 4 grading categories based on genetic, molecular and histological profiles ranging from Grade 1 (least severe) to Grade 4 (most severe) (10). Pediatric high-grade gliomas, which account for 8-12% of pediatric CNS tumors are classified as grade 3 and grade 4 tumors due to the inherent ability to rapidly proliferate and invade neighboring tissue from a pathophysiological perspective with genetic characteristics also taken into consideration (10, 11). Grade 3 pHGG are generally defined as malignant neoplasms displaying nuclear atypia capable of diffusely infiltrating surrounding healthy tissue to a greater extent than grade 1 and 2 pHGG (10). Grade 4 pHGG are significantly more malignant than grade 3 tumors, and frequently exhibit additional histological features such as florid microvascular proliferation (MVP) of blood vessels and pseudopalisading necrosis whereby necrotic tumor tissue is surrounded by hypercellular nuclei (12).

The most recent pHGG classification system defined by the WHO (WHO CNS 5) has outlined four subtypes as distinct categories within the pediatric subclass. These include “diffuse midline gliomas (H3 K27-altered)”, “diffuse hemispheric gliomas (H3 G34-mutant)”, “diffuse pediatric-type high-grade gliomas (H3-wildtype and IDH-wildtype)” and “infant type hemispheric gliomas (ALK, MET, NTRK 1/2/3 or ROS1 fusion)” (10). While no definitive conclusions have been made yet, it is widely believed that diffuse midline gliomas originate from oligodendroglial progenitor cells (13, 14). The genetic profiling of wildtype and mutant variants of genes such as isocitrate dehydrogenase 1 and 2 (IDH1/2), histone H3, anaplastic lymphoma kinase (ALK), MET proto-oncogene (MET), neurotrophic tyrosine receptor kinase (NTRK) and ROS proto-oncogene 1 (ROS1) to name a few are essential for a definitive diagnosis (10). Unlike prior classifications, a clinically advantageous diagnostic distinction which will aid disease management has been introduced to present adult high-grade gliomas (aHGG) and pHGGs as separate entities, with the exception of ambiguous tumors which harbor overlapping features. The two distinct anatomical locations where pHGGs arise from are hemispheric and midline structures. Prior to the reclassification, anaplastic astrocytomas (AA) were the most common grade 3 pHGG diagnosed in children (15). However, the descriptor “anaplastic” is no longer used as all tumors previously labelled as anaplastic were defined as grade 3 tumors despite preexisting clinical differences in disease progression. Tumors diagnosed prior to the criteria update will likely be recategorized retrospectively. Other subclasses of grade 3 HGG such as oligodendrogliomas, gangliogliomas and pleomorphic xanthoastrocytomas exist, but are extremely rare and often poorly characterised in pediatric oncology (10, 16, 17). Glioblastoma multiforme (GBM) has been the most prevalent form of grade 4 pHGG and has an extremely poor 5-year survival rate of 1.2% (18). However as of the 2021 classification update, the term glioblastoma is no longer diagnostically used and these tumors will likely be recategorized as diffuse hemispheric gliomas (DHG) or diffuse pediatric-type high grade gliomas (10). Additional subclasses of pHGG such as diffuse midline gliomas (DMG) (previously named diffuse intrinsic pontine gliomas or DIPG) of midline structures and gliosarcoma (glial and sarcomatous tumor) exist, but have previously been clinically regarded as separate entities. With the advent of updated standards, it is highly likely that many aforementioned tumor types will undergo retrospective reclassification in concordance with the WHO 2021 criteria by assessing genetic, histological and molecular characteristics.

Patients affected by pHGG may display various symptoms and signs such as ataxia, headaches, diplopia, papilledema, seizures, speech impediments, auditory impairment, behavioral changes, difficulties balancing and vomiting (18). However, such symptoms are not unique to the disease and are not independently diagnostic. Although the precise etiology of pHGG is largely unknown, a multitude of different factors can increase patient predisposition. Exposure to ionizing radiation emitted from an energy source intentionally as a form of prior cancer treatment or incidentally from the environment, can destabilize electrons within atoms and consequently induce cellular mutations and promote tumorigenesis (19). However, the true initiation of this disease is hypothesized to be multifactorial as the extent of the possible influence from factors such as pathogens, toxins, medication, cigarette smoke and background radiation to name a few, have remained inconsistent on their own (20). Cancer predisposition syndromes (CPS), responsible for germline mutations within specific genes can elevate a patient’s risk of developing pHGG (21). Three primary cancer predisposition syndromes known as Constitutional mismatch repair deficiency (CMMRD), Li-Fraumeni syndrome (LFS) and Neurofibromatosis-1 (NF-1) are some of many CPS which increase susceptibility to pHGG as a subsequent somatic mutation can initiate the onset of disease. CMMRD is a syndrome whereby biallelic mutations in at least one of the four mismatch repair genes MLH1, MSH2, MSH6 and PMS2 results in the inability of cells to repair incorrectly copied DNA sequences during DNA replication (22, 23). The subsequent accumulation of mutations can predispose carriers of the deficiency to cancer (22–24). Li-Fraumeni syndrome is a CPS whereby at least 75% of patients harbor an autosomal-dominant mutation of the tumor suppressor gene TP53, pivotal to the transcription of tumor protein 53 (p53) which regulates the cell cycle (25). Forty one percent of LFS patients have developed tumors by the age of 18 years (25). NF-1 is a condition caused by a germline mutation of the NF1 gene responsible for producing neurofibromin (21). Neurofibromin is a tumor suppressor protein which regulates cell proliferation by suppressing Ras, a protein responsible for the promotion of cell proliferation, adhesion and differentiation. Patients with NF-1 experience abnormally elevated levels of cell growth due to Ras activation and are at an increased risk of developing malignancies (26). Despite such risk factors, the true initiation of pHGG tumorigenesis for individual cases remains unresolved. While it is difficult to ascertain the cause of pHGGs, thorough cross-examination of patient lifestyle choices, environmental and geographical exposures, diet and previous medical procedures to name a few, may prove beneficial for harnessing conclusive findings.

## 2 Current approaches to treatment

Patients presenting with the symptoms previously outlined will generally be diagnosed with pHGG following a combination of diagnostic procedures. A computed tomography (CT) scan, magnetic resonance imaging (MRI) or both are performed for visual confirmation. The diagnosis is finalized using a biopsy in conjunction with a lumbar puncture to assess potential tumorigenic infiltration of the cerebrospinal fluid (CSF). MRI scan results or potentially liquid biopsies may be used as the final diagnostic tool in certain cases of the midline and brainstem where surgery is not possible nor worth the risk (27, 28). Patients diagnosed with pHGG will traditionally undergo a combination of surgery, radiotherapy and chemotherapy depending on suitability (29). Suitable pHGG patients may directly or following recurrence, be appointed to clinical trials for more targeted forms of therapy dependent on the stage and progression of disease.

### 2.1 Surgical resection

Surgical resection serves an integral role in correctly diagnosing a tumor by obtaining sufficient tissue for histopathological analysis in conjunction with DNA methylation and Omics studies. The extent of tumor resection, when possible, is case specific and dependent on the localization and infiltration to surrounding tissue. Tumors occurring within midline or infratentorial structures, or those that have diffused into vital structures are prime examples which are difficult to surgically ablate (30). The degree of tumor removal can generally be classified as gross total resection (GTR) (100%), near total resection (>90%), sub-total resection (51- 90%) or partial resection (10-50%) (31). The Children’s Cancer Group (CCG)-945 study conducted on a cohort of pHGG patients diagnosed with AA and GBM revealed radical resection of a tumor (more than 90%) resulted in a significantly greater five-year progression-free survival (PFS) (44 ± 11% and 26 ± 9% respectively) when compared against less radical resection (22 ± 6% and 4 ± 3% respectively), highlighting the positive significance of resecting maximal tumor volume prior to treatment (31). Unsurprisingly, many studies acknowledge surgical resection as the leading prognostic indicator of overall patient survival (32–34). A recent study has placed significant value on surgical expertise by identifying the extent of tumor ablation during the first surgical procedure as being the leading determinant of overall survival, as subsequent surgical procedures provided no significant improvement in patients when compared against those who did not undergo GTR (35). Such epidemiological studies may benefit by considering the influence of established pHGG subtypes (WHO CNS 5), as certain mutations will often occur concurrently in specific regions of the brain and possibly explain certain statistical patterns. Unfortunately, surgery presents risks such as the possibility of infection, intracranial hemorrhage, blood loss induced hypovolemia and neurological deficits to name a few (36). Although the degree of total tumor resection is considered as a leading prognostic indicator, the highly heterogenous nature of pHGGs, and variations between tumor localization and infiltration of healthy tissue often means surgical resection is insufficient on its own (32, 37). Thus, surgery should be considered as one pivotal step in a complex multimodal treatment protocol.

### 2.2 Radiotherapy

Radiation therapy is used in pHGG patients to eliminate residual traces of a tumor following surgery or as a primary protocol in inoperable cases of the midline. However, its usage is avoided in patients under the age of 3 years due to the potential neurocognitive harm that can occur in the developing brain which experiences pivotal milestones of cognitive development during this time (38, 39). Such children are at an increased risk of developing radiotherapy related complications such as leukoencephalopathy, stunted bone development and intellectual disabilities (40–43). Conventional radiotherapy where 54 Gy of radiation is delivered in several doses (1.8 Gy per dose) over a 6-week period, particularly in inoperable pHGGs, has often been used as a standard for treatment alongside adjuvant chemotherapy to prolong patient survival (44). Radiotherapy both alone and as part of a multi-treatment regimen consisting of surgery and chemotherapy does yield an increased overall survival with the latter being the conventional clinical recommendation. Furthermore, the reirradiation of recurrent tumors which have previously undergone radiation therapy yields an increase in overall survival, with a retrospective study conducted on 40 supratentorial pHGGs revealing a significantly greater median survival time of 9.4 months in reirradiated patients as opposed to 3.8 months in those who were not (45). While reirradiation does yield a better overall prognosis for pediatric patients, a signature population of radiation induced gliomas defined as secondary malignancies originating within previously irradiated regions with histology dissimilar to the original tumor and no evidence of CPS have also been studied and present a clinical conundrum (19, 46–48). Findings from the Childhood Cancer Survivor Study revealed that children exposed to radiotherapy were at risk of developing neoplasms with children exposed at ages 5 or less being at the highest risk which may highlight the increased vulnerability of the developing brain to radiation induced mutagenesis (19). One study found secondary brain tumors appeared more commonly in patients that underwent primary cranial radiation at doses greater than 25 Gy but additional data may be necessary due to the small sample size (49). However, as is the case with many therapeutic strategies, the emergence of resistance is a barrier which must often be overcome and in the case of radiation therapy, agents acknowledged as radiosensitizers have been examined to assess the ability to reintroduce sensitivity towards radiotherapy or to enhance pre-existing efficacy (50). Targeting pathways involving Notch, poly-ADP ribose polymerase (PARP) and mammalian target of rapamycin (mTOR) to name a few, concomitantly with radiotherapy, have been shown to enhance the efficacy of radiation therapy (51–53). However, despite promising results, there has been no significant improvement in pHGG patient survival from a clinical perspective. Although methods enhancing the efficacy of radiotherapy are essential, the risks associated with radiotherapy remain unchanged for the time being and highlight a necessity to establish safer and more efficacious treatment strategies in the long term.

### 2.3 Chemotherapy

Chemotherapeutic compounds are often administered to pHGG patients as an adjuvant treatment either intrathecally, intraventricularly, intravenously, or orally when surgery and radiotherapy are deemed insufficient to prevent disease progression or when patients are too young for radiation therapy. Temozolomide (TMZ) is a chemotherapeutic drug approved for use against malignant astrocytomas and GBMs with one of its primary characteristics being the rare ability to cross the BBB (54). TMZ acts as an alkylating and methylating agent which binds to DNA to impede cell proliferation. The administration of TMZ has been considered as a standard of treatment in conjunction with radiotherapy and surgery since the publication of a landmark study in adult GBM revealing an improvement in overall survival (54). Additional findings in pediatric cases have accentuated the significance of concurrent and adjuvant TMZ in prolonging patient survival with numerous clinical trials in place (55). While utilizing clinical therapeutic strategies deemed effective in aHGG is beneficial in certain cases, one must discern aHGG and pHGG as clinically distinct subtypes harboring genetic and molecular differences. Consequently, a greater emphasis should be placed upon therapies targeted against specific pediatric molecular signatures. While TMZ itself does present therapeutic value, its effects are predominantly pronounced within MGMT (O-6-methylguanine-DNA methyltransferase) methylated tumors as opposed to unmethylated tumors (median overall survival (OS) 24.59 months and 14.11 months respectively) (56). As MGMT methylation is comparatively rare in pHGG when compared with aHGG, greater clinical success is likely to be found elsewhere through therapeutics targeting pediatric-centric aberrations (57). Other chemotherapeutic compounds such as carboplatin (alkylating agent), irinotecan (topoisomerase inhibitor), lomustine (alkylating agent), vincristine (vinca alkaloid) and vorinostat (histone deacetylase inhibitor) are also subject to being part of adjuvant therapies against pHGG although none have yielded any significant improvements in overall patient survival and the prognosis remains dismal. Overall, the prevalence of heterogenous subtypes within the broader pHGG cohort highlights the importance and impending likelihood of clinicians diverging away from generalized treatment protocols riddled with off-target side effects, towards personalized subtype specific therapies targeting intra-tumoral characteristics.

### 2.4 Limitations and difficulties of treating pHGG

**Figure 1:**
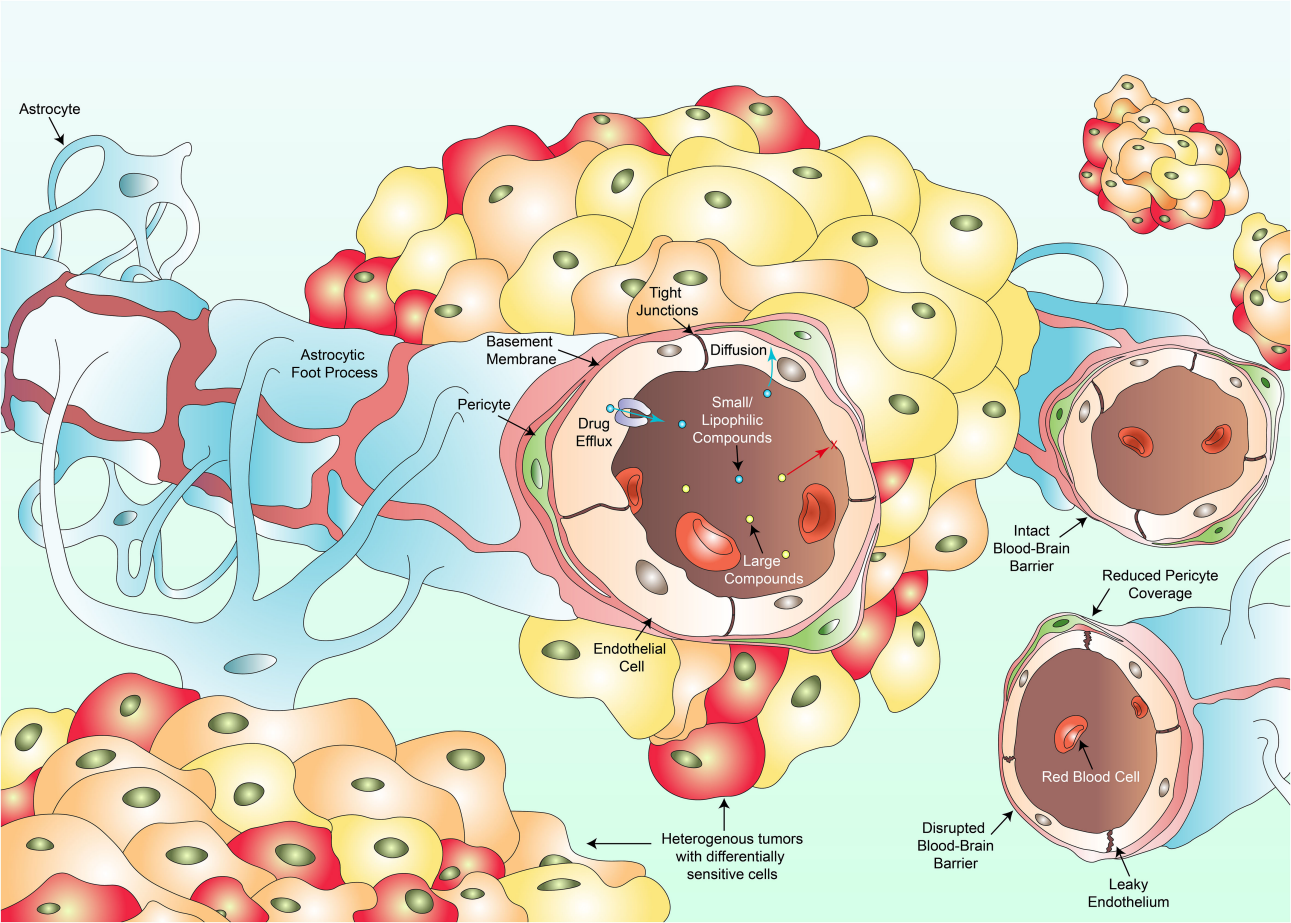
Several key factors hinder the clinical success against pHGG. Intra-tumoral heterogeneity culminates in individual cell populations within a tumor harboring differential drug sensitivities varying from resistant to hypersensitive responses (multi-colored cancer cells). The existence of the blood-brain barrier composed of endothelial cells, tight junctions, pericytes, the basement membrane and astrocytes, prevents large compounds (yellow dots) from crossing towards the site of the tumor. Only small or lipophilic compounds (blue dots) can passively diffuse through. Even such compounds which have diffused through may be pumped back into circulation via drug efflux transporters. Vascular heterogeneity results in blood vessels portraying varying degrees of permeability to therapeutics. Some regions of the BBB remain intact, a feature more commonly observed in DMGs whilst other regions are disrupted with a leaky wall, disrupted tight junctions and a reduction in pericyte coverage. Such disruptions are more common in cortical pHGGs. Variations in permeability lead to fluctuations in regional tumor perfusion and therapeutic exposure to a drug.

Several key factors heighten pHGG’s status as being one of the most difficult childhood malignancies to treat (**Figure 1**). Tumor heterogeneity from both intra-tumoral and inter-tumoral perspectives present the need to therapeutically account for genetic, molecular and histopathological variations within a patient’s tumor and among larger patient cohorts (8, 58). Such is the degree of inter-tumoral heterogeneity among the pediatric population, that the 2021 WHO CNS classification has accounted for different subtypes consisting of histone H3 K27 and G34R/V variants, IDH1 and IDH2, ALK, NTRK, MET and ROS1 to name a few due to variations in overall survival, site of origin and therapeutic response (10). Additional tumor subtypes highlighting inter-tumoral heterogeneity, affecting TP53, receptor tyrosine kinase (RTK), ATRX chromatin remodeler (ATRX) and MYCN to name a few, occur within unique pediatric subpopulations. These mutated pathways differ in their molecular and subsequent clinical impacts when driving tumorigenesis as will be discussed later in this review and thus require tailored approaches in the clinical management of individual tumors. Intra-tumoral heterogeneity arising within an individual patient’s tumor with varying genomic and phenotypic profiles due to clonal variation has also been observed. This accentuates the importance of combinatorial therapy and precision medicine to account for differences in treatment sensitivity and resistance which are likely to arise among unique clonal subpopulations of a tumor (58).

Moreover, clinical progress has been hampered by the presence of the BBB which serves a primary role as an endothelial barrier protecting the brain from circulatory plasma content, toxins and xenobiotics (59). Most cancers diagnosed annually including skin, lung, prostate, colorectal, breast and liver cancer to name a few, share commonality as treatment does not need to overcome the BBB (60). Such clinicians aim to ensure that therapies do not pass into unwanted sites such as the brain. Consequently, research into the establishment of BBB penetrating compounds occur to a lesser extent than nonpenetrating drugs. Diseases of the CNS would holistically benefit from preferential research conducted in this field. Not only must systemically administered therapeutics contend with a barrier preferentially selective for smaller or lipophilic compounds, but drugs which have successfully crossed the BBB may be actively transported back out via efflux pumps such as p-glycoprotein, rendering the drug therapeutically void in that specific instance (61, 62). Alternative therapeutic approaches are currently being investigated to bypass the BBB and will be covered later in this review. The BBB itself also displays heterogeneity (63). A recent study revealed significant heterogeneity within the BBB and its associated vasculature when comparing cortical pHGGs and DMGs, with indications of variation existing in response to extrinsic signals generally found within the tumor microenvironment (64). Cortical gliomas displayed significantly more irregularities in angiogenesis and BBB function in comparison to DMGs which maintained an intact BBB. Further research investigating variations in the functional integrity of the BBB between different pHGG subtypes prior to and during treatment may be clinically beneficial in not only understanding disease progression, but also developing novel therapeutics and methods for delivery.

Although the significance of current treatment protocols for pHGG are undeniable, the underlying side effects and poor relative long-term survival rates accentuate the need to investigate more novel targeted therapeutic approaches. Advancing the understanding of mechanisms driving tumorigenesis is insufficient on its own and must progress alongside the development of novel therapeutic approaches to target these mechanisms. Combining this approach with novel techniques to improve treatment efficiency will likely yield significant improvements in overall patient survival.

## 3 Alternative avenues for treatment

With overall prognosis and long-term survival rates remaining poor, researchers are delving into alternative treatment avenues to overcome current obstacles. The necessity for alternative approaches to delivery is exemplified further by findings from one specific study which highlighted that while H3K27M mutant tumors (discussed later in this review) have a worse overall survival rate than H3 wildtype tumors of the same location, H3K27 wildtype and mutant tumors with diffuse tumor characteristics have a worse prognostic outcome than non-diffuse tumors of both H3 wildtype and mutant status (65). This burden placed by tumor location regardless of mutant status can only be overcome through the optimisation of current strategies in place for delivering therapeutics and the discovery of novel therapeutic approaches.

### 3.1 Convection enhanced delivery

Convection enhanced delivery is one such method involving the stereotactic insertion of a catheter to enable the delivery of treatment directly to the interstitial fluid beyond the BBB, near the site of the tumor with minimal systemic exposure (66, 67). Clinical CED relies on the principle of bulk flow whereby a solution flows down a pressure gradient provided by an infusion pump. While treatment is still affected by structures within the brain tissue itself, this method enables clinicians to circumvent the high doses often required for local therapeutic affect at the intended site. This method does face limitations including the potential backflow of the infused drug into nontargeted regions of the brain resulting in reduced dose exposure, drug efflux, heterogeneous intra-tumoral vasculature capable of distributing treatment to other sites, and alternating intra-tumoral pressure due to oedema (68, 69). However, the method’s ability to overcome the BBB and increase tumor exposure to therapeutic doses is undeniable. Studies within pHGGs have showcased promising results for dose exposure, which if combined with subtype-specific strategies, may yield favorable outcomes (70).

### 3.2 Chronotherapy

Chronotherapy, a novel strategy involving the utilization of the circadian clock to dictate treatment timing, has demonstrated that administering compounds at night-time increases BBB permeability due to gap junction mediated reductions of intracellular magnesium essential for efflux (71). Studies performed using rhodamine B (RHB) as a tracer dye revealed intravenous injection resulted in the highest levels within the brain immediately after awakening with minimal efflux (71). Despite its clinical relevance being unknown in pediatric patients and its utilization potentially being quite difficult in younger patients with inconsistent sleep cycles, chronotherapy may provide a novel avenue for optimizing the efficacy of pre-existing drugs at significantly lower concentrations to eliminate toxicity while enhancing the therapeutic capacity of compounds discovered in the future. Immunotherapy may be one such approach to benefit from chronotherapy with research increasingly hinting at the immune system being circadian regulated with the time of treatment influencing overall survival (72). Investigations into artificially manipulating local factors associated with circadian rhythm may also be beneficial for administering drugs outside the hours discussed in these studies.

### 3.3 Immunotherapy

Utilizing the immune system to eradicate pHGG via targeted immunotherapy is another treatment strategy which may be of great benefit in the future (**Table 1**).

**Table 1:** Ongoing clinical trials in the field of immunotherapy targeting pHGG.

Immunotherapy Strategy	Ongoing Clinical Trial Identifier	Status	Phase	Therapeutic Entity
CAR T-cell	NCT05438368	Recruiting	1 and 2	bi-4SCAR-GD2/CD70
	NCT05835687	Recruiting	1	B7-H3-CAR T
	NCT03638167	Active, not recruiting	1	EGFR806 CAR T
	NCT04185038	Recruiting	1	B7-H3-CAR T
	NCT05768880	Recruiting	1	B7-H3, EGFR806, HER2, IL13-Zetakine CAR T
	NCT04099797	Recruiting	1	GD2-C7R CAR T
	NCT05544526	Recruiting	1	GD2 CAR T
	NCT04196413	Recruiting	1	GD2 CAR T
Monoclonal Antibody	NCT03389802	Active, not recruiting	1	APX005M (CD40 humanized mAB)
Antibody-Drug Conjugate	Currently no clinical trials	N/A	N/A	N/A
Vaccine	NCT04749641	Recruiting	1	H3.3-K27M targeted neoantigen peptide
	NCT01130077	Active, not recruiting	1	HLA-A2-Restricted Glioma Antigen-Peptides with Poly-ICLC
	NCT03299309	Active, not recruiting	1	PEP-CMV
Oncolytic Virus	NCT02457845	Active, not recruiting	1	G207 Oncolytic HSV
	NCT05717712	Recruiting	1	Ad-TD-nsIL12
Immune Checkpoint Inhibitor	NCT02359565	Recruiting	1	Pembrolizumab (PD-1 mAB)
	NCT04323046	Active, not recruiting	1	Nivolumab (PD-1 mAB), Ipilimumab (CTLA-4 mAB)

Chimeric antigen receptor T-cells (CAR T-cells) are an immunotherapeutic strategy harboring the potential to transform the therapeutic landscape of pHGG medicine. DNA artificially synthesized to express cancer-specific antigen receptors are inserted into patient-derived T-cells in a laboratory (73). Millions of such modified CAR T-cells harboring the ability to recognize and specifically bind to cancer associated antigens are generated and infused back into the patient to eradicate malignant cells. Unfortunately, success has predominantly been limited to hematological malignancies (74, 75). However, studies have recently alluded to therapeutic success in pHGGs. CAR T-cells targeting the disialoganglioside GD2 which is highly expressed in H3K27M-mutant DMGs have demonstrated robust efficacy both *in vitro* and in orthotopically transplanted xenograft models (76). The first clinical study of its kind translating these findings in pediatric H3K27M-mutant DIPG and DMG patients, has showcased specific efficacy towards K27M-mutant cells with prolonged patient survival (77). Further studies expounding upon the CAR T-cell-tumor axis and addressing tumor heterogeneity through the identification of alternative targetable antigens are necessary to facilitate increases in overall patient survival, as no patients survived long term. Although its main function is largely speculative, B7-H3 (CD276), a co-stimulatory molecule responsible for T-cell recruitment, has recently been implicated in the development of pediatric CNS tumors (78, 79). CAR T-cells targeting B7-H3 activity in xenograft models of CNS tumors have showcased enhanced efficacy relative to tumors expressing low B7-H3 antigen levels, of which similarly low levels are also observed in normal tissue (79). Preliminary findings from the first human phase 1 clinical trial assessing B7-H3 CAR T-cell efficacy in recurrent CNS tumors and DIPGs (NCT04185038), has demonstrated dose tolerability in conjunction with sustained clinical improvements in certain patients, necessitating further experiments (80). Ongoing pHGG CAR-T trials are focused on GD2, B7-H3, EGFR806, HER2 and IL13 (**Table 1**). Identifying novel subtype specific antigens by utilizing Omics data should yield more targeted subtype specific therapeutic responses in the foreseeable future.

Antibody drug conjugates (ADC) are an emerging class of pharmaceuticals combining the cytotoxicity of small molecules with the accuracy and precision of immunotherapy (81). Scientific breakthroughs over the past few decades have culminated in third generation ADCs comprised of a fully humanized antibody with high binding affinity for a tumor-specific surface antigen harboring little to no expression on healthy tissue (82, 83). A cytotoxic molecule is bound to this antibody via a stabilized chemical linker which releases the molecule intracellularly or extracellularly upon binding to the site-specific antigen. This humanized antibody prevents an unwarranted anti-ADC immune response, while circumventing the risk of conjugate-related off-target effects by mediating site specificity. Furthermore, the capacity of one antibody to carry multiple entities highlights an opportunity for combinatorial therapy. While studies have been conducted in aHGG using anti-EGFR monoclonal antibody (mAB) bound ADCs such as AMG-595 (NCT01475006) and ABT-414 (NCT01800695, NCT02343406, NCT02573324, NCT02590263), no clinical trials have been conducted to date in pediatric patients (84). Furthermore, EGFRvIII is more commonly altered in adults as opposed to pHGG. Clinical approaches incorporating ADCs and other antigen associated immunotherapies will likely see greater progress through investigations into pHGG predominant antigen expression. A recent study has revealed the potential role of anti-interleukin 13 receptor subunit alpha 2 (IL13Rα2) related ADCs as a candidate via a subset of therapeutically hypersensitive DIPG cell lines (85). As specific antigens such as GD2, EPHA2 and B7-H3 have been found with key tumorigenic roles in pHGG, investigations uncovering the immunogenic landscape of pHGGs may prove fruitful for ADC development.

Personalized cancer vaccines are another branch of immunotherapy which prime the immune system to recognize tumor-derived neoantigens and drive an antitumorigenic response within an inherently immunosuppressive environment. Vaccine delivery may be classified as cell-based, nucleic acid-based, peptide-based or virus-based (86, 87). Peptide-based vaccines comprised of a polypeptide construct mimicking known or predicted neoantigens are the most common method investigated in pHGG with multiple ongoing clinical trials (NCT04749641, NCT01130077, NCT03299309). Researchers investigating a H3.3K27M specific neoantigen (NCT04749641) in a cohort of DIPG patients, have thus far described a mutation specific upregulation in CD4^+^ and CD8^+^ T-cells, with minimal adverse side effects and a median progression free survival of 11.7 months (still increasing) (88). No patients have experienced disease progression. Dendritic cell (DC) vaccines are the most common cell-based vaccine and are synthesized using patient-derived monocytes which are matured into dendritic cells (89). DCs are pulsed with neoantigens which are consequently presented as epitopes on the DC surface, ready for patient injection. There are currently no clinical trials underway for pHGG DC vaccines. The administration of nucleic acid vaccines harboring patient derived tumor RNA and DNA have also arisen as therapeutic possibilities. Neoantigen screening may revolutionize the development of novel subtype specific vaccines by addressing unique aberrations (90, 91). Vaccines may additionally benefit through the incorporation of a multi-neoantigen approach.

Oncolytic viruses are a class of antitumorigenic viral therapeutics which selectively lyse tumor cells and disrupt tumor microvasculature, while simultaneously evoking an immunostimulatory response to counteract tumor-driven immunosuppression (92, 93). Following the approval of the first oncolytic virus in 2005 (H101 adenovirus), viral vectors including adenoviruses, coxsackie viruses, herpes viruses, measles viruses, New Castle disease viruses, polioviruses, poxviruses and reoviruses have been investigated for oncolytic activity (92, 94). Oncolytic viruses are best delivered intratumorally due to the presence of the BBB. Following favorable results in adults, the genetically engineered adenovirus Delta-24-RGD (DNX-2401) was administered in pHGG and DIPG mouse models, resulting in a significant increase in survival (95). The resultant clinical trial driven by this study (NCT03178032) on 12 newly diagnosed DIPG patients, revealed a reduction in tumor size in 9 patients, partial response in 3 patients and disease stabilization in 8 patients, albeit with adverse side effects (96). Another oncolytic virus G207, a genetically modified herpes simplex virus type-1 (HSV-1), has demonstrated a greater degree of efficacy in pHGG relative to aHGG (97). An ongoing G207 phase 1 clinical trial (NCT02457845) in supratentorial pHGG tumors has thus far demonstrated a marked increase in lymphocytic tumor infiltration and acceptable risk profiles (98).

Immune checkpoints (IC) negatively regulate the immune system by inactivating or diminishing the extent of an activated immune response to preserve self-tolerance. Aberrant IC activity has been implicated in the progression of tumors through immune surveillance blockade. Investigations utilizing IC inhibitors in patients with hypermutant pHGGs harboring biallelic mismatch repair deficiency delivered favorable outcomes (99). Although there are several checkpoint inhibitors targeting PD-1 (pembrolizumab, nivolumab) and CTLA-4 (ipilimumab) under clinical investigation for pHGG, results thus far have been underwhelming and cohorts may benefit from a stratified subgroup-based approach.

### 3.4 Focused ultrasound stimulated microbubbles

Focused ultrasound (FUS) stimulated intravascular microbubbles present another therapeutic strategy through which BBB permeability is transiently increased via sonoporation to efficiently deliver therapeutic compounds to the tumor (100). In this method, acoustic pressure is applied via a transducer to sonicate specific regions of the brain (101–103). Microbubbles comprised of phospholipid microspheres containing an inert gas are intravenously injected alongside a therapeutic entity. As these microbubbles pass through the FUS waves, they vibrate, mechanically disrupting the local endothelial cell wall by opening tight junctions. This temporary disruption to the BBB allows the passage of therapeutics into the site of the tumor. Furthermore, FUS has shown potential in temporarily suppressing p-glycoprotein expression with the period of suppression dependent on settings used during experimentation (104). While clinical trials are currently limited to adults, FUS stimulated microbubbles present an interesting non-invasive therapeutic strategy for enhancing the bioavailability of preexisting and futuristic therapies at the tumor site. Ultrasound devices intracranially implanted during tumor resection are also under clinical investigation in adult GBM (NCT04528680) (105).

### 3.5 Sonodynamic therapy

Sonodynamic therapy is a novel therapeutic strategy that has recently emerged in pHGG as a non-invasive option with minimal adverse side effects. SDT involves the delivery of a nontoxic sonosensitizer compound, which upon accumulation within the tumor, is activated via ultrasound to elicit localized cytotoxicity by reactive oxygen species (ROS) production (106). The sonosensitizer 5-aminolevulinic acid (5-ALA) has preferentially emerged in SDT due to its selective affinity for HGG tissue (107). 5-ALA is metabolized into protoporphyrin IX (PPIX) which selectively accumulates within tumor tissue prior to ultrasound mediated activation and ROS production (108). The first and only pediatric SDT clinical trial (NCT05123534) which began in late 2022 aims to investigate the impact of 5-ALA SDT in DIPG (109). Photodynamic therapy (PDT) from which SDT was inspired, uses light as opposed to ultrasound but is less preferred due to the limited penetration of tissue by light which requires invasive procedures to be as effective in HGG (110). Ultimately, SDT provides a diffuse non-invasive solution for holistically scoping the entire brain to eradicate elusive tumors which characterize the diffuse nature of HGG.

### 3.6 Tumor treating fields

Tumor treating fields present a unique non-invasive approach to treating brain tumors, by utilizing alternating electric fields to target mitotic proteins within mitotic cells to induce cell cycle arrest and subsequent cell death (111). TTFields have been approved for use in adult GBM since 2015 following findings from the EF-11 and EF-14 trials (112, 113). The EF-11 study, the first of its kind conducted on an adult cohort of recurrent GBM (aged 23-80) revealed that although the difference in 6-month progression free-survival was insignificant between TTFields and chemotherapy alone (21.4% and 15.1%), TTFields offered a greater quality of life (112). The EF-14 trial revealed a significant increase in overall survival when TTFields were delivered in combination with TMZ (20.5 months) as opposed to TMZ alone (15.6 months) (113). However, 80% of study participants relapsed regardless of treatment at 18 months and despite its benefits in combination with traditional forms of treatment, further research with optimisation is necessary to enhance the impacts of TTFields. TTFields have not been approved for use in pediatric cohorts yet with clinical trials solely focused on adults highlighting its value as a potential treatment with minimal cytotoxicity (114, 115). Pulsed electric fields have also been implicated *in vitro* and *in vivo* with transiently opening the BBB and may be suitable for enhancing drug bioavailability (116–118).

### 3.7 Therapy-loaded nanocarriers

Nanocarriers capable of carrying therapies within or in a conjugated state have emerged as novel strategic candidates to efficiently transfer therapeutic compounds with enhanced bioavailability across the BBB into the tumor. Particularly when aided by the strategies previously outlined. Nanocarriers include nanoliposomes, phospholipid drug conjugates, micelles, dendrimers, carbon dots, SPIONs and EDVs to name a few (**Table 2**). The exterior surface of such nanocarriers can be modified to evade premature phagocytic digestion and enhance tumor specificity using surface conjugates such as tumor specific ligands, antibodies, aptamers, transferrin, and peptides (142, 143). Despite favorable outcomes *in vivo*, there are no ongoing clinical trials for pHGG, particularly when compared to adults, highlighting largely uncharted territory with therapeutic promise. However, it is pivotal that investigations are conducted to definitively ascertain the absence of unwarranted lingering side effects post-nanocarrier treatment.

**Table 2:** Nanocarrier technology with the potential to enhance tumor perfusion and the bioavailability of therapies at the tumor site.

Nanocarrier	Description	Able to Cross the BBB?	In Vivo or Human Brain Tumor Studies for Nanocarrier-Drug Conjugates *
Nanoliposome	A therapeutic entity is encapsulated within a phospholipid bilayer which is released into the tumor cells via endocytosis	Yes	Jhaveri et al. (2018) (119) Ashrafzadeh et al. (2020) (120) Grafals-Ruiz et al. (2020) (121) Zheng et al. (2020) (122)
Phospholipid Drug Conjugate (PDC)	A therapeutic warhead bound to a phospholipid ether (PLE) attaches to the surface receptors of the tumor. Upon the accumulation of multiple PDCs on the external membrane, the drugs are cytoplasmically internalised via transmembrane flipping.	Yes	Iopofosine i-131 (patented product by Cellectar Biosciences)
Micelle	Micelles are single layer amphipathic monospheres comprised of a hydrophobic core ideal for carrying hydrophobic compounds. Drugs are released by endocytotic degradation of the micelle or targeted diffusion following stimulation by light, temperature, pH etc.	Yes	Xu et al. (2021) (123) Zhang et al. (2022) (124)
Dendrimer	Dendrimers are radially branched symmetrical constructs comprised of a central functional core with branched oligosaccharides, peptides and glycopeptides encapsulating a drug of choice. The outer branches can be modified to be either hydrophobic or hydrophilic and may also hold a drug. Drugs are released following endocytosis.	Yes	Xu et al. (2016) (125) Sharma et al. (2018) (126) Sharma et al. (2020) (127)
Carbon dots	Carbon dots are carbon nanoparticle suspensions composed of a hybridized carbon core from which functional groups extend and to which drug conjugates are attached.	Yes	Liyanage et al. (2020) (128) Li et al. (2020) (129) Li et al. (2021) (130) Muhammad et al. (2022) (131)
Superparamagnetic iron oxide nanoparticles (SPION)	SPIONs are magnetic field guided nanoparticles comprised of an organic or inorganically coated maghemite, magnetite or hematite core. A drug of choice can be loaded within or conjugated to the surface.	Yes	Xu et al. (2016) (132) Ganipineni et al. (2018) (133) Afzalipour et al. (2019) (134) Patel et al. (2023) (135)
EnGeneIC Dream Vector (EDV)/ Armed Nanocell Drug Conjugate (ANDC)	EDVs or ANDCs are a non-living nanocell system derived from non-pathogenic *Salmonella typhimurium* bacteria. The nanocell loaded with a therapeutic, binds to cancer cells via a bispecific antibody. The EDV is internalised by the cell and the drug released intracellularly.	Yes	Solomon et al. (2015) (136) Whittle et al. (2015) (137) MacDiarmid et al. (2016) (138) Kwan et al. (2018) (139) Sagnella et al. (2020) (140) Khan et al. (2021) (141)

*There are currently no ongoing clinical trials listed for pHGG according to ClinicalTrials.gov.

## 4 Addressing the tumorigenic landscape of pHGGs

### 4.1 Histone modifications

The past decade has revealed pHGG to be significantly distinct from its adult counterpart. One particularly distinguishing factor involving the discovery of substitution mutations within the H3F3A and HIST1H3B genes, which encode the H3.3 and H3.1 histones respectively (144, 145). These mutations predominate pediatric populations with rare exceptions diagnosed in adults. Histones reside within a stacked octamer harboring duplicate copies of H2A, H2B, H3 and H4 histones, wrapped in 147 base pairs of DNA to form a nucleosome (146). This enables the dense packing of DNA into chromatin and is tightly regulated through acetylation, methylation, phosphorylation and ubiquitination during transcription and cell replication (147, 148). Aberrations of the histone tail have been implicated in driving underlying oncogenic changes of the epigenome. As of 2021, the profound tumorigenic impact of histones has resulted in 2 of the 4 pHGG subtypes being classified according to their histone status (10). Namely, diffuse midline gliomas (H3 K27-altered) and diffuse hemispheric gliomas (H3 G34-mutant).

The H3 mutation H3K27M is one of 2 primary mutations identified in pHGGs, with a point mutation at the 27th codon (AAG to ATG) resulting in the substitution of lysine (K) with methionine (M) (149). The polycomb repressive complex 2 (PRC2) methyltransferase acts preferentially towards H3K27 to methylate H3K27me1 (monomethylation), H3K27me2 (dimethylation) and H3K27me3 (trimethylation) (150, 151). Enhancer of zeste homolog 2 (EZH2) acts as PRC2’s catalytic subunit, mediating the methylation of H3K27 into H3K27me3, which in turn silences gene expression and maintains native cell identity and lineage (152). Dysregulated interactions between polycomb-group (PCG) members and their target genes can have tumorigenic consequences (153). Mutant H3K27M has been associated with a global reduction in H3K27me3 expression in pHGG and is hypothesized to inhibit PRC2’s EZH2 domain from catalyzing the methylation of H3K27me3 (154–156). Structural analysis has revealed that mutant H3K27M binds to the SET-domain of EZH2 with an affinity 16-fold higher than wildtype H3K27 to assert an oncogenic inhibitory role upon PRC2 (157). Despite mutant K27M being hypothesized to sequester PRC2 and impair EZH2 release, consequently reducing H3K27me3; conflicting evidence has shown that the proportion of chromatin bound EZH2 remains unchanged in H3.3K27M (155, 158). This is due to the transient nature of H3K27M-PRC2 interactions which are complemented by lasting inhibition of PRC2’s enzymatic activity long after dissociation, culminating in reduced H3K27me3 expression (159). Post-translational modifications proximally and distally to H3 histone’s K27 residue can reduce the extent of K27M mediated PRC2 inhibition, highlighting a therapeutic vulnerability (160). The presence of H3K27M mutations have also been accompanied by a global reciprocal increase in H3K27M acetylation and H3K4me3, consequently elevating gene transcription (161–163). The complexity of this mutation may be better understood by identifying the functions of interactions between K27M-associated molecules and neighboring histone residues. Removing H3K27M expression has been shown to restore H3K27me3 and eradicate tumor burden, accentuating the reversible nature and potential therapeutic targetability of this mutation (156). Developing compounds to competitively disrupt the interaction between the PRC2 complex’s EZH2-SET domain and mutant H3K27M while maintaining uninterrupted PRC2 conformational activity may be efficacious. Tazemetostat is an EZH2 inhibitor currently under clinical trial in a generalized pediatric cohort harboring mutant EZH2 (NCT03155620). While EZH2 mutations are rare in the context of H3K27M tumors, tazemetostat may be of interest for clinicians to evaluate its efficacy in managing H3K27M activity.

A comprehensive study conducted upon 1030 pHGG data samples revealed H3.3K27M mutations of the H3F3A gene to be the most frequent subgroup of histone mutants accounting for 30% (316) of total cases (164). Initially thought to be exclusive to pediatric cohorts, H3.3K27M is most frequent in young children, with the mean age of diagnosis being 9.4 years (165). However, recent studies have conversely revealed H3K37M to also be a potential driver in rare cases of aHGG (166). Pediatric H3.3K27M mutations account for 63% of DIPG and 59.7% of midline gliomas associated with the brainstem, cerebellum, thalamus and spine (164). In contrast, H3.1K27M tumors which are restricted to the pons, arise much earlier (median age of 5), while being comparatively less common than the H3.3K27M variants (164). Patients diagnosed with H3.3K27M pHGG experience a shorter survival expectancy of 11 months as opposed to the slightly prolonged 15 months observed in H3.1K27M patients. Both mutations have a significantly less favorable prognosis relative to H3K27 wildtype tumors (164). Additional mutations can also selectively co-occur alongside distinct histone mutations. The receptor tyrosine kinase fibroblast growth factor receptor 1 (FGFR1), undergoes fusion or mutates in a subset of thalamic H3.3K27M gliomas (167). The presence of H3.1K27M in midline tumors of the pons may be accompanied by upregulated Activin-A Receptor Type 1 (ACVR1) expression, a protein with proliferative and metastatic capacity implicated in numerous cancers (167). Importantly, ACVR1 mutant diseases have previously been responsive to the retinoic receptor agonist palovarotene, with efficacy repeated in a small cohort of DMG patients, whereby the one patient harboring an ACVR1 mutation experienced tumor stabilization for 30 weeks (168). However, more data is necessary to definitively substantiate this finding. These mutations highlight underlying cellular interactions essential to tumor growth which may present targetable therapeutic opportunities. A small molecule dopamine receptor D2 (DRD2) antagonist and imipridone ONC201 has shown promising results in small scale clinical trials against H3K27M-mutant pHGGs, with studies also revealing a role in targeting pHGG cell metabolism (169, 170). As the exact mechanisms driving H3K27M-mediated tumorigenesis become better understood, therapeutics designed to accordingly target such factors with subtype specificity will likely enhance clinical efficacy.

The second histone mutation identified in pHGG, H3G34R/V, also occurs at the H3 histone but involves a point mutation at the 34th codon, substituting glycine (G) with arginine (R) or valine (V) (144). H3G34R/V mutations are restricted to the H3F3A gene, solely existing as H3.3 mutants (145). Unlike their H3K27 counterpart, comparatively fewer details are known about H3G34 mutations. H3G34R/V mutations prevent methylation of the nearby H3K36 residue due to the bulky nature of the amino acids replacing the smallest known amino acid glycine (171). Consequently, the extended side chains prevent the histone methyltransferase SET domain-containing 2 (SETD2) from binding to *cis* H3K36 to methylate H3K36me3, which is essential for recruiting mismatch repair (MMR) protein MutSα (172–174). G34R/V mutations are implicated in MMR deficiency-induced tumorigenesis. One study revealed G34R tumor-bearing mice exhibiting downregulations in DNA-damage response (DDR) pathways, to be selectively sensitive to PARP (pamiparib) and cell-cycle (AZD7762) inhibitors (175). H3G34R-centric experiments have demonstrated histone lysine demethylase subfamily 4 (KDM4) to preferentially bind to the mutant histone and abrogate its demethylase activity, resulting in a global increase in H3K36me3 (176). Similar globally upregulated levels have been observed in G34V-mutant tumors, highlighting H3K36me3 as a potential transcriptional activator of downstream oncogenic pathways (177). Such loci may allude to hypothetically alternative therapeutic targets for pHGG. Larger sample sizes will amplify the ability to confidently characterize these complex molecular profiles, as external regulatory factors may also be of influence. H3K27me3 enrichment has also been observed in H3G34V-mutant cells (174). Zinc Finger MYND-Type Containing 11 (ZMYND11), a tumor suppressor which elicits its regulatory mechanisms through H3K36me3 identification is inhibited in the presence of mutant H3G34 (178). While no therapeutic compounds exist to date, targeting the interactions outlined above, with a key focus on H3K36me3 associated downstream signaling pathways, may enhance clinical success.

H3G34 mutant pHGGs arise within supratentorial and hemispheric regions of the brain, accounting for 16.2% of cortical tumors with a greater tendency to arise in the parietal and temporal lobe; contrary to K27M mutants which occupy the pons and midline structures (164). A recent study incorporating 257 H3G34-mutant pHGGs revealed G34V mutant tumors to harbor a worse overall prognosis than G34R mutants (median OS of 9.9 and 14.8 months respectively) (179). Variations among factors associated with tumor diffusion or underlying downstream pathways may explain such discrepancies. The median age of diagnosis is higher than that of K27M-variants at 15 years, emphasizing its prevalence in older pediatric patients (164). This same study found the median OS to be 18 months with disease progression being less severe than that of H3K27M. Variations between the long-term survival rates of G34R/V and K27M mutants can be attributed to key observable factors such as the hemispheric nature of G34R/V mutant tumors providing better surgical access for tumor resection.

One study has revealed NOTCH1 inhibition via DAPT, a γ-secretase inhibitor which prevents the cleavage of Notch1, to selectively sensitize H3.3 mutant cell lines to cell death to a significantly greater degree than wildtype histone variants (180). While there are currently no therapeutics to specifically target these histone mutants directly, the aforementioned mechanisms in conjunction with co-occurring mutations offer viable therapeutic targets. These include H3.1K27M with ACVR1 as well as H3.3G34R/V with ATRX, PDGFRA and TP53 which may provide additional subtype specific avenues for combinatorial therapy (164, 179).

### 4.2 Tumor protein 53

The TP53 gene encoding tumor suppressor protein 53, serves a pivotal role in tumor suppression by regulating apoptosis, autophagy, cell-cycle arrest and senescence (181). Mutations driving the somatic inactivation of TP53 and consequential upregulation of oncogenic activity have been observed across most pHGGs (164, 182–184). TP53 mutations have been associated with a significant increase in clinical resistance to radiotherapy (185). TP53 mutations co-segregate with H3.3K27M mutations to enable apoptotic evasion and are observed in up to 77% of pediatric DIPGs (184). Studies have revealed the incidence of mutant TP53 to be as high as 78% in H3.3K27M and H3.3G34R/V positive HGG and 75% of H3-wildtype HGG (184). Furthermore, 54% of supratentorial cases of GBM in pediatric patients have been found to exhibit TP53 inactivation (184). Despite its high prevalence in pediatric cohorts, TP53 loss-of-function mutations are significantly more frequent in patients over the age of 3 (40%) as opposed to younger patients (11%) (186). This may be indicative of TP53 requiring a second somatic hit acquired during the patient’s lifetime, to induce the onset of disease in a manner parallel to that observed in CPS.

Recurrent point mutations of the TP53 DNA-binding domain resulting in protein instability allude to increased tumorigenesis in pHGG being driven by altered downstream signaling pathways (187). The influence of TP53 on other pathways is further exemplified by findings from a study conducted across 854 pHGG tumor samples revealing a significant association between the presence of TP53 mutations and high somatic structural variant (SSV) burden involving large scale genomic alterations (188). Gain-of-function mutations within protein phosphatase, Mg^2+^/Mn^2+^ dependent 1D (PPM1D), a wildtype p53-induced phosphatase 1 (Wip1), are another TP53 pathway-associated mediator of tumorigenesis which act independently of TP53 status to inhibit p53 activity (189). PPM1D mutations were observed in 37.5% of samples in a cohort comprised of brain stem gliomas whereby TP53 mutations and PPM1D were mutually exclusive (190). Consequently, TP53 and PPM1D mutations are widely accepted as having similar dysfunctional impacts on p53 activity. PPM1D can be therapeutically targeted using small molecule inhibitors. One such study conducted upon DIPGs unveiled the reactivation of DNA damage response pathways which are usually inhibited in the presence of mutant PPM1D (191). P53 activity is also controlled by the protein byproducts of genes mouse double minute 2 homolog (MDM2) and mouse double minute 4 (MDM4). Mdm2 negatively regulates p53 activity as an E3-ubiquitin protein ligase by driving p53 towards proteasomal degradation, while Mdm4 negatively regulates p53 activity by binding to the p53 transcriptional activation domain. Enhanced Mdm2 activity is observed in pHGG alongside reduced p53 expression levels promoting tumorigenesis and presents another viable therapeutic opportunity (192).

While targeting pathways associated with p53 expression such as PPM1D, Mdm2 and Mdm4, do elicit TP53 dysfunction-dependent therapeutically beneficial outcomes, TP53 as a lone entity, has remained therapeutically elusive (193). Although its incidence across a multitude of pHGG subtypes makes it an ideal therapeutic candidate, variability induced by the dynamic conformational state of mutant TP53 has complicated drug development (194). Eprenetapopt (APR-246) has emerged as one of the first reactivators of mutant p53, mediating its activity by binding to the cysteine residues to thermodynamically stabilize p53 in its functional conformation and initiate apoptosis (195). A new class of p53 inhibitors capitalizing on these findings may revolutionize the clinical landscape. Two futuristic approaches with therapeutic potential involve gene therapy and the stabilization of mutant TP53 activity using peptides to restore native TP53 activity. The transfection of tumor-derived cell lines with functional wildtype p53 has been shown through multiple studies to induce cell senescence and apoptosis (192, 196). Researchers have also demonstrated unique methods utilizing CRISPR-Cas9 to detect and kill p53-deficient tumor cells (197). However, the delivery of such transformative vectors will require delivery methods which circumvent the BBB, with site specificity, to avoid off-target effects and ensure adequate tumor perfusion. Peptide-based therapies using lead peptides to stabilize wildtype p53 activity have induced regression of tumor growth in mouse xenograft models with researchers believing that p53 is in a constant state of dynamic equilibrium (198). Thus, when mutated p53 proteins adopt the wildtype conformation, these peptides may be stabilizing and increasing the proportion of correctly folded p53. Despite being an evidently sound concept for alleviating TP53-driven tumorigenesis, such therapies have attendant toxicities which require a deeper mechanistic understanding. Studies directed towards understanding the structural behavior and implications of mutant TP53 may fast-track the development of therapies for what is currently a clinically elusive aberration.

### 4.3 Receptor tyrosine kinase pathways

Receptor tyrosine kinase pathways, in conjunction with phosphoinositide 3-kinase (PI3K) activity, play a fundamental role in a subset of pHGGs, albeit differently to the mechanisms observed in aHGGs due to significant genomic discrepancies. RTKs are a class of transmembrane receptors with intrinsic enzymatic activity initiated by the binding of extra-cellular signaling molecules. This ligand-receptor complex mediates the dimerization and subsequent phosphorylation of neighboring RTKs, activating tyrosine kinases which facilitate the activity of downstream pathways such as PI3K. While native RTK activity encompasses cell-to-cell interactions, differentiation, maturation, metabolism and motility to name a few, aberrant RTK activity is known to be a significant tumorigenic driver implicated in cancer cell survival and proliferation (199). Two predominant families of RTKs established as pivotal in HGG tumorigenesis are the RTK class I epidermal growth factor receptor (EGFR) family and RTK class III platelet derived growth factor receptor (PDGFR) family (11, 164). Aberrant PDGFR activity is especially significant in pHGG.

PDGFR is well established for its importance in maintaining healthy cellular activity throughout the body. It accounts for two subunits PDGFRα and PDGFRβ encoded by the PDGFRA and PDGFRB genes respectively, which co-exist as either twin pairs or in combination on the cell membrane to mediate downstream signaling once bound by a PDGF ligand (200). Despite the exact mechanisms behind its involvement in tumorigenesis remaining unknown, aberrations in PDGFRA have been implicated in the progression of numerous diseases such as cancer, representing the most mutated RTK in pHGG (11, 164, 201). Furthermore, PDGFRA overexpression has even been observed in the absence of amplifications (202). Aberrant PDGFRA expression is observed in both pHGG (29.3%) and aHGG (20.9%) to a significant degree (203). A large-scale genomic study conducted on 290 patients revealed 18.3% of pHGGs including AA, DIPGs and GBMs to be harboring PDGFRA amplifications (7.2%), mutations (9.0%) or both (2.1%) (excluding patients outside conventional pediatric age parameters) (204). PDGFRA amplifications were predominantly observed within the DIPG subset (62%) while PDGFRA mutations were conversely found more often within hemispheric GBMs (54%) (204). A recent study revealed 15% of pHGGs to be harboring PDGFRA alterations, of which H3K27M DMGs exhibited significantly higher levels of PDGFRA, highlighting a targetable locus (205). Another study has shown that 50% of hemispheric H3.3G34R/V mutations harbor a PDGFRA mutation (201). PDGFRA mutations were more prevalent in the older pHGG cohort (mean age of 14.5 years) when compared to the non-mutant population (mean age of 9.4 years) (204). The co-occurrence of G34R/V and PDGFRA mutations in an older population may possibly allude to an alternative origin for this subset of tumors harboring differential lineage-specific characteristics. Both mutant and amplified PDGFRA is associated with poor overall prognosis in hemispheric pHGG, when analyzed using Kaplan-Meier analysis (204). Although its prevalence is comparatively lower in aHGG, adult tumors harboring PDGFRA amplification have maintained a worse prognostic outcome relative to wildtype tumors (203). Furthermore, the phosphorylation of downstream targets of PDGFRA in the absence of a binding ligand has showcased the presence of constitutive activity for mutant PDGFRA (202).

Unlike histone and TP53 mutations, PDGFRA inhibitors do exist. Therapeutically targeting PDGFRA using inhibitors such as dasatinib and crenolanib have shown moderate efficacy although the modes of action appear to predominantly be cytostatic, not cytotoxic (202). As a result, combinatorial therapy in conjunction with PDGFRA inhibition may offer a more viable solution for tumor regression. Avapritinib, a BBB penetrating PDGFR inhibitor, is currently being investigated as a potential therapeutic against PDGFRA mutant pHGG and has shown favorable toxicity profiles (205–208). Adult GBM cell lines of the proneural subtype have shown sensitivity to PDGFRA inhibition (209). Adopting a stratified subtype specific approach should enable the identification of anti-PDGFRA hyper-responsive pHGGs, upon whom therapies would be more efficacious. Unlike pHGGs, aHGGs are significantly more reliant on the activity of EGFR. Up to 50% of adult GBMs display aberrant amplification or overexpression, emphasizing the importance of addressing aHGG and pHGG as individual entities (210).

PI3Ks are a family of lipid kinases pivotal to native cellular activity. However, PI3K activity is considered a hallmark in the progression of numerous cancers including pHGG and aHGG, with a consequentially poorer prognosis (211, 212). PI3Ks exist as specific classes of which class IA are activated by RTKs such as PDGFRA. Class IA PI3Ks comprised of p110α, p110β and p110δ interact with the p85 subunit (213). The p110α subunit then mediates phosphatidylinositol-4,5-bisphosphate (PIP2) conversion to phosphatidylinositol-3,4,5-triphosphate (PIP3) (213). This allows phosphoinositide-dependent kinase 1 (PDK1) to dock and phosphorylate Akt to mediate downstream tumorigenic activity (213). Mutations within this pathway have been documented in pHGGs. Mutations of the PIK3CA gene which encodes the p110α subunit are associated with increased PI3K activity and have been identified in 21% of pHGGs, 15% of DIPGs and 17% of aHGG (211, 212). Mutations within p110β and p110δ have also been identified to a lesser extent. The tumor suppressor gene phosphatase and tensin homolog (PTEN) is responsible for negatively regulating PI3K activity via the conversion of PIP3 back to PIP2 (213). Loss-of-function mutations result in PIP3 overexpression and subsequent increases in PI3K pathway activation. Despite its high prevalence in aHGG (25-50%), PTEN mutants represent a significantly small subset of pHGGs at 1-5% (214). PTEN mutations are associated with a poorer prognosis regardless of age (215). PI3K-inhibitor paxalisib is currently under clinical investigation in a subset of DMG (NCT05009992). Combinations of MEK and PI3K inhibitors have also showcased synergistic efficacy (205, 216, 217). Interestingly, the synergistic activities of upstream activator RTKs and downstream signaling molecules of the PI3K pathway highlight a therapeutic opportunity for combinatorial therapy in suitable pHGG patients.

### 4.4 MYCN

The MYCN proto-oncogene from the MYC family of regulatory genes is also associated with aberrant activity in a subset of pHGG (164). MYCN serves as a transcription factor to regulate downstream activity of pro and anti-apoptotic mechanisms, embryonic development, cell proliferation and metabolism through DNA sequence-specific binding mechanisms (218). A study aiming to characterize H3/IDH-wildtype pHGGs, identified 3 unique categories comprised of PDGFR amplified RTK I, EGFR amplified RTK II and MYCN amplified tumors (219). The MYCN group represented the highest frequency of cases (41%) and poorest overall survival (14 months) (219). The WHO has acknowledged these subtypes in its 2021 rendition classifying CNS tumors under the H3-wildtype and IDH-wildtype category for diffuse pediatric-type high-grade gliomas. MYCN mutant and amplified tumors are primarily hemispheric (83.8% MYCN positive cases) of which most are temporal (43.2%) while a minor subset is thalamic (13.5%) (220). Unlike the previous study, this study did not find MYCN-associated tumors to have a significantly worse overall survival compared to the RTK I subtype. This may be due to variations in sample size and the potential co-occurrence of confounding mutations which were not the primary focus of either study and present a point worthy of exploration (220). Although both supratentorial and infratentorial MYCN-amplified tumors are shown to be of similar molecular and histopathological status, their hemispheric predominance may be indicative of lineage specific mechanisms which differ between these two anatomical regions (220). Furthermore, MYCN amplifications seldom occur alongside H3.3K27 mutant tumors (221). H3.3G34 mutations which predominate hemispheric tumors, experience a significant upregulation of MYCN in the absence of gene amplification, highlighting another hypothetical subtype specific therapeutic route (177). MYCN cannot be targeted directly from a clinical standpoint yet due to the undefined nature of its transcription factor binding pockets. Nonetheless, numerous mechanisms indirectly targeting this pathway, including the use of aurora kinase A (AURKA) and bromodomain and extra-terminal motif (BET) inhibitors, to name a few, provide viable therapeutic opportunities to decrease MYCN expression and reduce tumor volume (222). Only one clinical trial is currently in place with respect to aberrant MYCN-expressing pediatric tumors (NCT03936465), highlighting a substantial void in the clinical management of this subtype with therapeutics targeting MYCN alone or in combination.

### 4.5 ATRX

The ATRX chromatin remodeler (ATRX) gene encodes the ATRX protein which belongs to the Switch/Sucrose Nonfermentable 2 (SWI/SNF2) family of ATP-dependent chromatin remodeling helicases; classified as such due to the helicase/ATPase domain located at the C-terminus (223). Naturally, ATRX forms a complex with H3.3 histone chaperone death-associated protein 6 (DAXX) to initiate H3.3 deposition into pericentromeric heterochromatin and the telomeres to facilitate chromatin remodeling and regulate transcription (223, 224). ATRX functions as a tumor suppressor and thus, loss-of-function mutations at the ATRX locus reduce H3.3 deposition, and facilitate telomeric instability and subsequent homologous recombination to initiate telomerase-independent alternative lengthening of telomeres (ALT) (225). Aberrant ATRX activity commonly occurs alongside K27 and G34 mutations of H3.3. Mutations associated with H3.3 ATRX chromatin remodeling pathways have been found in 44-70% of pHGGs with a much lower prevalence of 9% in DIPGs (144, 184, 226). One study revealed, 31% of pHGGs to harbor mutations in both ATRX and DAXX with 100% of G34R/V mutations shown to overlap with this cohort highlighting a lineage-specific co-dependency between G34 mutant tumors and ATRX status (144). As observed in G34-mutant pHGGs, ATRX mutations also occur in older pediatric patients (mean ages 11-17) suggestive of ATRX loss having an age specific influence on tumor progression (184). Mutations of ATRX co-occur alongside TP53 mutations; an observation which may be explained by ATRX loss on its own being insufficient to induce tumorigenesis due to apoptotic resolution while loss of both ATRX and TP53 promotes tumorigenic phenotypes (144, 227, 228). Consequently, the ATRX loss-of-function mutation and its tendency to cooccur alongside TP53 and H3.3 mutations to promote genomic instability, may highlight co-dependencies of therapeutic significance. Although ATRX cannot be directly targeted using small molecules, ATRX deficient pHGGs have demonstrated selective sensitivity towards PARP inhibitors such as olaparib, rucaparib and talazoparib (229). ATRX-deficient HGGs have also shown enhanced sensitivity towards RTK and PDGFR inhibitors (230). Investigations establishing the tumorigenic mechanisms behind the loss of ATRX activity, telomeric attrition and site-specific structural analysis of ATRX-mediated histone deposition may bring forth novel therapeutic solutions.

### 4.6 IDH

Mutations of isocitrate dehydrogenases 1 (IDH1) and less frequently 2 (IDH2), key metabolic enzymes involved in the citric acid cycle, have been identified in a subset of pHGG (231, 232). IDH1 and IDH2 drive the oxidative decarboxylation of isocitrate to α-ketoglutarate and carbon dioxide within the cytosol and mitochondria respectively. Mutant IDH1 and IDH2 undergo a conformational change resulting in enzymatic activity which converts α-ketoglutarate to its structural derivative 2-hydroxyglutarate which has been found at high concentrations in mutant gliomas (232, 233). IDH mutant tumors are commonly found in secondary aHGGs and are comparatively rare in pHGGs (234, 235). One study found 16.3% of a pediatric cohort to harbor an IDH1 mutation and no IDH2 cases, of which all cases arose within adolescents aged over 14 years (235). This may be indicative of a potential overlap between older pHGG and secondary aHGG characteristics. Conversely, IDH mutations arise more often in low-grade gliomas (LGG) and may be pivotal in the early stages of a malignancy prior to high-grade progression (234). Pediatric studies have demonstrated that IDH mutant tumors harbor a better overall prognosis relative to wildtype tumors (overall 1 year survival of 100% and 81% respectively) (235). Conflicting studies in adults have revealed that the long-term prognostic expectation may not be due to IDH, but rather additional underlying mechanisms, particularly for surviving beyond 3 years (236). The WHO CNS 5 classification has acknowledged IDH expression status as a signature diagnostic biomarker for subtyping. IDH-mutant proteins provide a druggable target with clinicians concluding that inhibitors such as the recently FDA-approved ivosidenib (IDH1 inhibitor) deserve further investigation (237). Virtual screens have revealed multiple druggable loci for IDH1 (238). Tumors expressing 2-hydroxyglutarate have shown greater sensitivity to PARP inhibitors in comparison to wildtype tumors, highlighting key IDH-subtype specific therapeutic dependencies (239).

### 4.7 BRAF

The BRAF oncogene which encodes the B-Raf protein, is a member of the RAF serine/threonine protein kinase family. It plays a pivotal role in cell differentiation and secretion, predominantly mediating downstream signaling activity through the MAPK/ERK signaling pathway (240). The most common BRAF mutation V600E, is found in a small subset of pHGG (approximately 5%) (241). It involves the substitution of the 600th residue valine with glutamic acid, resulting in the kinase domain of BRAF being 500-fold more active than native BRAF and driving constitutive downstream activation of the MAPK/ERK signaling pathway (241, 242). Importantly, studies have revealed BRAF V600E mutations to be more common within LGG which progressed into secondary pHGG (sHGG) (243). Although only 2.9% of HGGs in this study were secondary malignancies, 39% of these sHGGs harbored a BRAF V600E mutation (243). Moreover, all BRAF V600E mutations resided within the sHGG cohort. Cyclin-dependent kinase inhibitor 2A (CDKN2A) mutations were observed in 57% of these sHGGs with both mutations less common in LGGs which did not progress. As a result, BRAF V600E mutant sHGGs represent a unique subset of pHGGs which may benefit from synergistic therapies addressing mutant BRAF and its downstream MAPK/ERK signaling pathways. Both BRAF and its downstream target MEK are druggable, with BRAF inhibitors such as dabrafenib and vemurafenib showing promising results in BRAF V600E mutant tumors as either monotherapies or combinations alongside MEK inhibitors such as trametinib (241, 244, 245). This consequently warrants further subtype-specific clinical investigations.

### 4.8 Chromosomal instability and fusions

The cytogenetic landscape plays a focal role in the progression of pHGGs, with unique aberrations in chromosomal activity distinguishing pHGGs from aHGG (246). The most common pHGG changes involve gene fusions of NTRK, ALK, ROS1 and MET (all driven by RTKs), a gain of chromosome 1q, and loss of chromosome 4q (246–248). Tumors harboring NTRK, ALK, ROS1 and MET fusions have been classified in WHO CNS 5 under the umbrella term infant-type hemispheric gliomas due to their hemispheric predominance (96.7% of fusions) (247).

Fusions associated with NTRK, ALK and ROS1 have a 5-year overall survival of 42.9%, 53.8% and 25% respectively, with MET fusion positive tumors occurring to a comparatively lesser extent (247). The NTRK family consisting of NTRK1, NTRK2 and NTRK3 genes encode the tropomyosin receptor kinases (Trk) TrkA, TrkB and TrkC respectively, and are involved in normal brain activity and neurodevelopment (249). NTRK fusion genes encode a TRK protein, its catalytic tyrosine kinase domain and a fusion protein attached at the C-terminus which in combination can instigate oncogenic activity via downstream signaling pathways. A comprehensive study involving 127 pHGG cases revealed children under the age of 3 to most prominently harbor NTRK fusions, with 40% of such children harboring the alteration (250). Five signature NTRK fusion complexes including NTRK1 fusions with TPM3, NTRK2 fusions with VCL and AGBL4, and NTRK3 fusions with ETV6 and BTBD1 were identified in these tumors. NTRK fusions have been detected in 4% of DIPGs and 10% of non-brainstem HGGs (250). Clinical trials examining NTRK inhibitors entrectinib and larotrectinib are currently underway following successful preliminary studies which had shown favorable results in a pediatric context, with primary and metastatic tumors formerly deemed incurable experiencing significant regression (251–253). ALK fusions can be found in pediatric LGG, unlike NTRK, ROS1 and MET fusions which are pHGG-specific (247). Presenting in pHGGs of a lower mean age (1.6 months), ALK-driven pHGG can be targeted using therapeutics such as ceritinib, crizotinib, ensartinib and lorlatinib, although clinical data from a much larger cohort is paramount (247, 254). Lorlatinib appears to be especially promising having brought an ALK-fusion positive patient deemed incurable to a state of complete remission (254). Ensartinib has shown significantly greater efficacy in ALK positive non-small cell lung cancer patients when compared to crizotinib (25.8 and 12.7 months respectively) and clinical trials are currently in place for pHGGs harboring ALK, NTRK and ROS1 fusions (255). Capmatinib is another compound with clinical potential having induced significant reductions in tumor burden in patient-derived intracranial xenograft models harboring MET-fusions (256).

Genetic profiling comparing pHGG against aHGG has revealed key differences in the frequency for gain of chromosome 1q and loss of chromosome 4q (246). Gain of chromosome 1q has often pertained to a worse prognosis in cancers with elevated risks of recurrence (257). Gains in chromosome 1q are highly prevalent in 29% of pHGG and 30% of pediatric GBM while being significantly less common in adults (9%) (246). Another aberration commonly observed in pHGG (22%) as opposed to aHGG (2%) is a loss of chromosome 4q (246). Understanding the contribution of these cytogenetic aberrations to the progression of pHGG, is pivotal to decoding the influence such genetics have on the expressive capacity of downstream pathways. Especially if treatment is to be delivered accordingly.

## 5 The prospect of novel targeted therapy

**Figure 2:**
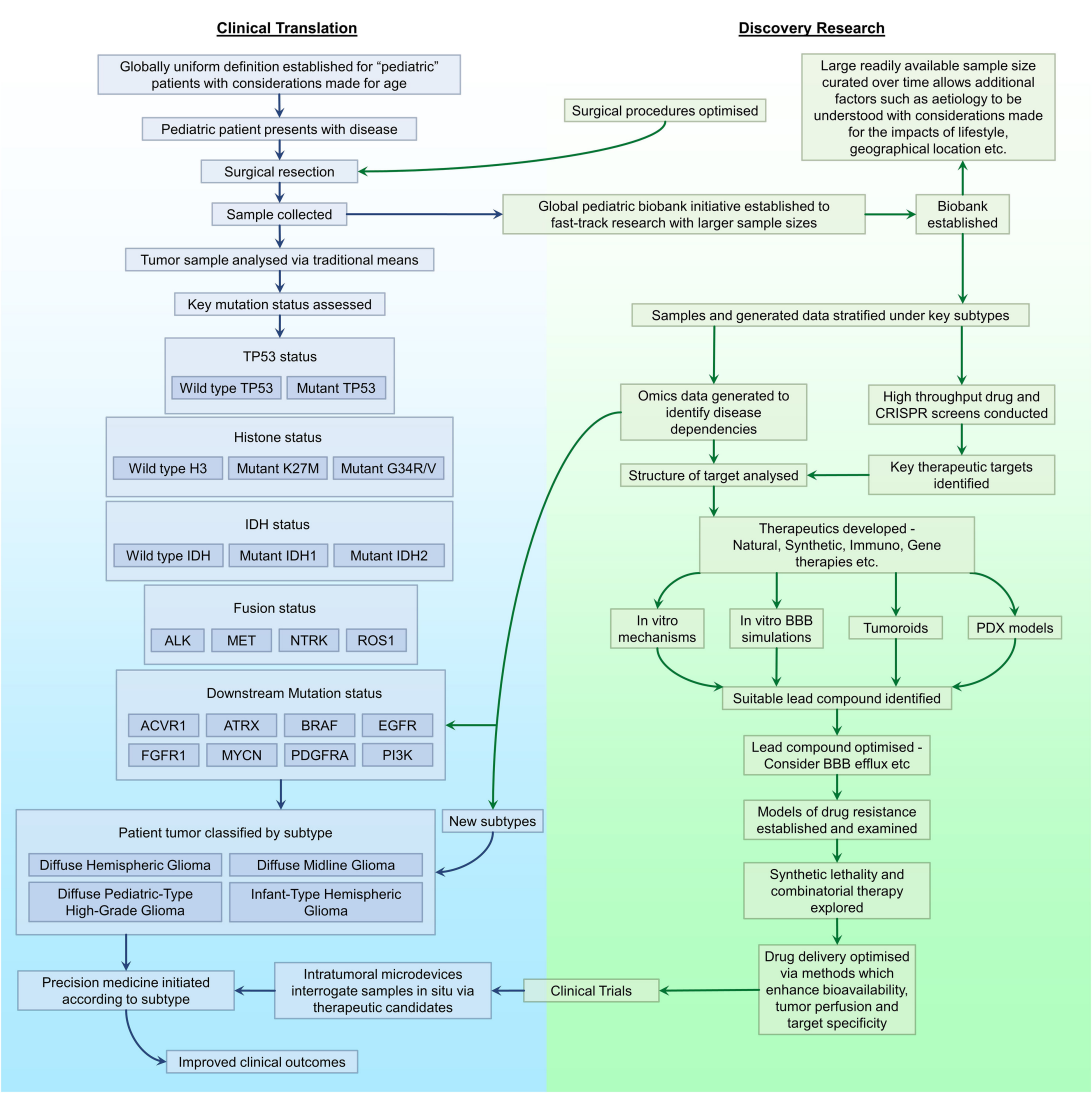
A proposed workflow for driving the progression of pHGG research. Outlined are key areas in need of improvement or optimisation in discovery research (green) and clinical translation (blue), and the association between each area with regards to the management of pHGG.

For decades, pHGGs have been the source of a clinical conundrum with the 5-year prognosis remaining poor. In the meantime, other childhood malignancies have conversely experienced significant improvements, a statistic which can be attributed to poor BBB penetrance and therapeutic perfusion of the tumor, intra and inter-tumoral heterogeneity and therapeutic resistance. Furthermore, the standard of care for pHGG has counterproductively mirrored that of its adult counterpart despite the distinct molecular, genomic, and epigenetic discrepancies between these two clinical entities. A fact formally acknowledged in the updated rendition of WHO CNS 5, capitalizing upon transformative research from the past decade exposing pHGG-centric subtypes. This decision will enable translational and clinical research to be conducted in a functionally targeted manner. Fortunately, such dismal prognoses are not due to pHGGs being objectively incurable. Hence, the dismal statistics can be overturned by altering the overarching approach to disease management, identifying novel dependencies underlying the progression of unique pHGG subtypes, establishing therapeutics to specifically target such dependencies, and adopting novel methods of delivery to enhance therapeutic perfusion of the tumor by said therapies (**Figure 2**).

As pHGGs account for a clinically rare malignancy, clinicians and researchers are met with a predominant issue regarding small sample sizes, often delaying the translational progression of potentially conclusive evidence. Although ambitious, one solution which would initiate transformative progress in this field, is the establishment of a global tissue biobank and data repository network connecting pre-existing and impending repositories in one accessible location (258, 259). This solution would provide researchers with greater access to globally derived samples which may otherwise remain inaccessible, to enable the progression of translational research with global clinical applicability. Not only will access to new data awaken the prospect of identifying novel tumorigenic mechanisms with statistical power, but the etiology of the disease may stand a better chance of being understood as confounding variables often differ by geographical proximity (i.e. lifestyle). Preventive recommendations may then be established as a societal norm. Furthermore, the stratification of data by variables such as tumor location, mutations, primary vs secondary tumors, radiation-induced gliomas, prior exposure to treatments and lifestyle factors would become more feasible. A global approach to pHGGs necessitates the harmonization of what is currently considered pediatric as the definition counterproductively varies between countries. One such solution would be to increase the age range from 0 to 22, with mutant status being the primary criterion for treatment as this is what drives the disease itself and would enable patients outside the age range to also be treated accordingly. Understandably, a global initiative regarding pHGGs would of course be complex and time consuming, as this entails considerations with regards to patient anonymity as well as logistic implications when managing and distributing tissue and data.

Precision medicine is a relatively new branch of medicine utilizing data gathered from an individual to design a patient-specific treatment protocol accounting for therapeutic susceptibility. With a high degree of unique characteristic variation existent within pHGG, this population would likely garner immense benefits from precision medicine with the potential for increased treatment efficacy, reductions in off-target side effects and an increase in prognosis and overall survival. Thorough approaches to the analysis of Omics data has resulted in the discovery of novel subtype-specific pHGG aberrations such as histone modifications H3.1K27M, H3.3K27M and H3.3G34R/V, gene fusions of NTRK, ALK, ROS1 and MET as well as abnormalities in TP53, PDGFRA, MYCN, ATRX, IDH and BRAF to name a few. As more data is acquired, novel tumorigenic abnormalities are likely to be discovered resulting in the formation of additional pHGG subtypes, enabling the continued exploration of therapeutically targetable dependencies. Subtype specific clinical strategies directly targeting tumorigenic loci in pHGGs are currently minimal with certain subtypes lacking any clinical trials (**Table 3**).

**Table 3:** Drugs of clinical relevance or potential with regards to efficacy against pHGG subtypes.

Drug	Aberrant Target	Direct or Indirect Target	Clinical Trial Identifier for pHGG-Specific Trials
ONC201	General pHGG efficacy (H3K27M preferred)	N/A (DRD2)	NCT04617002, NCT03416530, NCT03134131, NCT05009992
ONC206	General pHGG efficacy (H3K27M preferred)	N/A (DRD2)	NCT04541082, NCT04732065
Tazemetostat	H3K27M	Indirect (PRC2)	NCT03155620 (general cohort)
Palovarotene	H3.1K27M (ACVR1)	Indirect (Retinoic Receptor)	No trials
Pamiparib	H3G34R	Indirect (PARP)	No trials
AZD7762	H3G34R	Indirect (Checkpoint kinase)	No trials
Avapritinib	PDGFR	Direct (PDGFR)	NCT04773782
Paxalisib	PI3K	Direct (PI3K)	NCT05009992
BMS-986158	MYCN	Indirect (BET)	NCT03936465
Olaparib	ATRX	Indirect (PARP)	No trials
Rucaparib	ATRX	Indirect (PARP)	No trials
Talazoparib	ATRX	Indirect (PARP)	No trials
Ivosidenib	IDH1	Direct (IDH1)	No trials
Dabrafenib	BRAF	Direct (BRAF)	NCT04201457, NCT02684058, NCT03919071
Vemurafenib	BRAF	Direct (BRAF)	NCT01748149
Trametinib	BRAF	Indirect (MEK)	NCT04201457, NCT02684058, NCT03919071
Entrectinib	NTRK-fusion/ROS-1 fusion/ALK fusion	Direct (NTRK/ROS1/ALK)	NCT02650401
Larotrectinib	NTRK-fusion	Direct (NTRK)	NCT04655404, NCT02637687, NCT04945330
Ensartinib	ALK-fusion	Direct (ALK)	NCT03213652, NCT03155620
Lorlatinib	ALK-fusion/ROS-1 fusion	Direct (ALK/ROS1)	No trials
Capmatinib	MET-fusion	Direct (MET)	No trials

Intratumoral microdevices (IMD) are a novel device introduced recently for a first-in-human HGG clinical trial (NCT04135807) which may be of significant benefit in the field of precision medicine (260). IMDs are temporarily implanted during surgical resection, and nano-dosages of up to 20 individual drugs are administered to small regions of the tumor with surrounding tissue then extracted alongside the device. This approach enables *in situ* interrogation of drug-tumor interactions within the confines of an intact tumor to establish therapeutic efficacy, with exposed tissue subsequently available for downstream analysis. Participants have not exhibited any adverse effects.

The emergence of CRISPR (clustered regularly interspaced short palindromic repeats) technology capable of editing specific segments of a gene to alter activity has showcased its suitability for target discovery in a multitude of cancers. While CRISPR gene editing in its present state would not be suitable for direct applications in a clinical setting due to off-target side effects and *in situ* tumor accessibility, genome-wide CRISPR screens conducted in tumor samples *in vitro* and *in vivo* will likely expose tumor subtype specific dependencies in response to the alteration of specific genes. High throughput drug screens have proven to be equally valuable in not only identifying subtype vulnerabilities to specific classes of drugs but also provide an avenue by which drugs previously synthesized for non-cancer purposes can be repurposed for pHGG therapy (216, 261, 262). The cumulative data generated through Omics, genome wide CRISPR screens and high throughput drug screens will likely expose therapeutically targetable pathways unique to different pHGG subtypes and enable the synthesis of novel compounds (263).

A large majority of compounds are incapable of crossing the BBB to evoke any therapeutic response. Combining the vulnerable pathway specificity with considerations for BBB permeability and drug efflux mechanisms, should drive an increase in pHGG subtype specific compounds with enhanced clinical efficacy due to enhanced tumor perfusion. Furthermore, advances in *in vitro* models simulating the BBB may help streamline the ability to efficiently establish the capacity of a compound to elicit its intended effects prior to conducting time-consuming and complex large scale *in vivo* and clinical experiments (264). Establishing tumoroid models for pHGG, a field significantly lagging behind its adult counterpart, may also prove useful for recapitulating the tumor micro-environment, extracellular matrix, intra-tumoral dynamics and therapeutic responsivity (265). Pre-established patient derived xenograft (PDX) models comprised of immunodeficient mice implanted with patient-derived tumor samples for the multitude of established pHGG subtypes may provide an alternative method through which tumor specific vulnerabilities can be assessed to fast-track the clinical treatment of patients. However, one must understand that PDX models may not be the most accurate representation of endogenous human tumors due to variant selective pressures driving clonal selection prior to *in vivo* tumor establishment. Genetically modifying mice to harbor local gene knockouts for mutations which are known to concurrently drive tumorigenesis (i.e. H3.3K27M/TP53 loss/PDGFRA gain or TP53 loss/ATRX loss) may be another route through which pHGG onset can be mimicked *in vivo* (227, 266).

As tumor heterogeneity and drug resistance concomitantly impede clinical success, combinatorial therapy with a focus on synergistic drug combinations specific to unique pHGG subtypes will need to be investigated. Utilizing the concept of synthetic lethality, the mechanism by which the inhibition of two or more pathways is required to induce cell death as opposed to a single pathway alone, may also prove to be a viable option particularly with regards to H3K27M, H3G34R/V, TP53 and MYCN-mutant tumors which have generally remained elusive with regards to their direct therapeutic targetability. Synthetic lethality has previously been demonstrated in MYCN-mutant cell lines (267). As advances are made over upcoming years with regards to the identification of subtype specific targets, treatment targeting these pathways and methods of delivery to the sites of these tumors, precision medicine capitalizing on this knowledge in a clinical setting will likely see improved patient outcomes with increases in overall survival and quality of life.

## 6 Conclusion

Pediatric high-grade gliomas have presented a difficult class of tumors to treat, and the dismal 5-year survival rate emphasizes the need to modify current approaches to treatment. The standard protocol for treatment has remained unchanged for decades with surgery followed by adjuvant chemotherapy and radiation therapy still seen as the most suitable protocol. Although there is limited information on the etiology and origins of the disease itself, genomic, epigenomic and molecular advances have identified novel aberrations within histones H3K27 and H3G34, genomic alterations within TP53, PDGFRA, MYCN, ATRX, IDH to name a few and chromosomal alterations capable of driving tumorigenesis within individual subtypes of pHGG with differential sensitivities to individual therapeutics. WHO CNS 5 has acknowledged the significance of these findings by representing pHGG as a class separate from adult variants albeit with overlapping characteristics. Research has highlighted the need to better understand the progression of individual tumor subtypes to establish ideal therapeutic targets, particularly within tumors carrying mutations which cannot be directly targeted under current circumstances. The advent of precision medicine has spotlighted the necessity of developing therapeutics to target distinct subtype specific vulnerabilities with a primary focus on BBB permeability and optimized delivery mechanisms. Provided there is a significant increase in pediatric clinical studies, this paradigm shift in laboratory and clinical research settings to treating pHGGs, will likely yield significant improvements in patient survival and overall quality of life for this vulnerable population.

## Author contributions

DF: Writing – review & editing, Writing – original draft, Data curation, Conceptualization. AA: Writing – review & editing. BW: Writing – review & editing, Supervision, Resources, Project administration, Funding acquisition, Data curation.
